# Supplementary material for: ORAI1-Regulated Gene Expression in Breast Cancer Cells: Roles for STIM1 Binding, Calcium Influx and Transcription Factor Translocation
Source: Int J Mol Sci. 2022 May 24;23(11):5867. doi: 10.3390/ijms23115867 (PMC9180186; doi:10.3390/ijms23115867)
Supplement: Supplementary file 1 [file ijms-23-05867-s001.zip › ijms-1691100-supplementary.pdf]

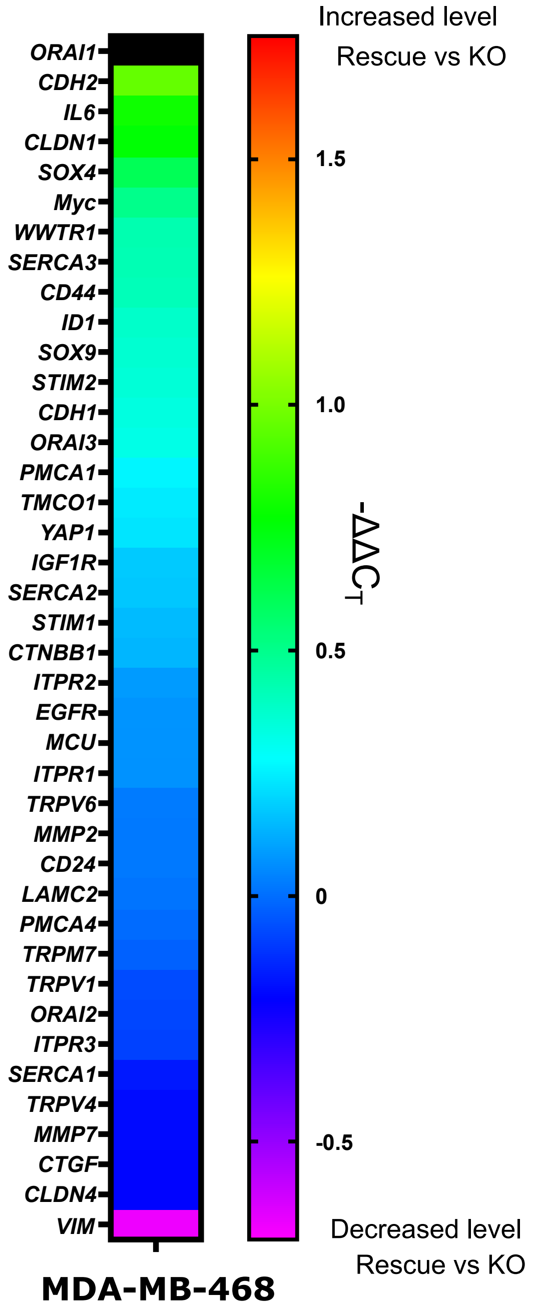

Supplementary Figure S1: ORAI1 regulated gene expression in MDA-MB-468 cells.

Heatmap representing extended gene list of mRNA $-\Delta\Delta C_T$ in MDA-MB-468 ORAI1 WT rescue compared to MDA-MB-468 ORAI1 knckout cells. Data represent the mean of three independent experiments.

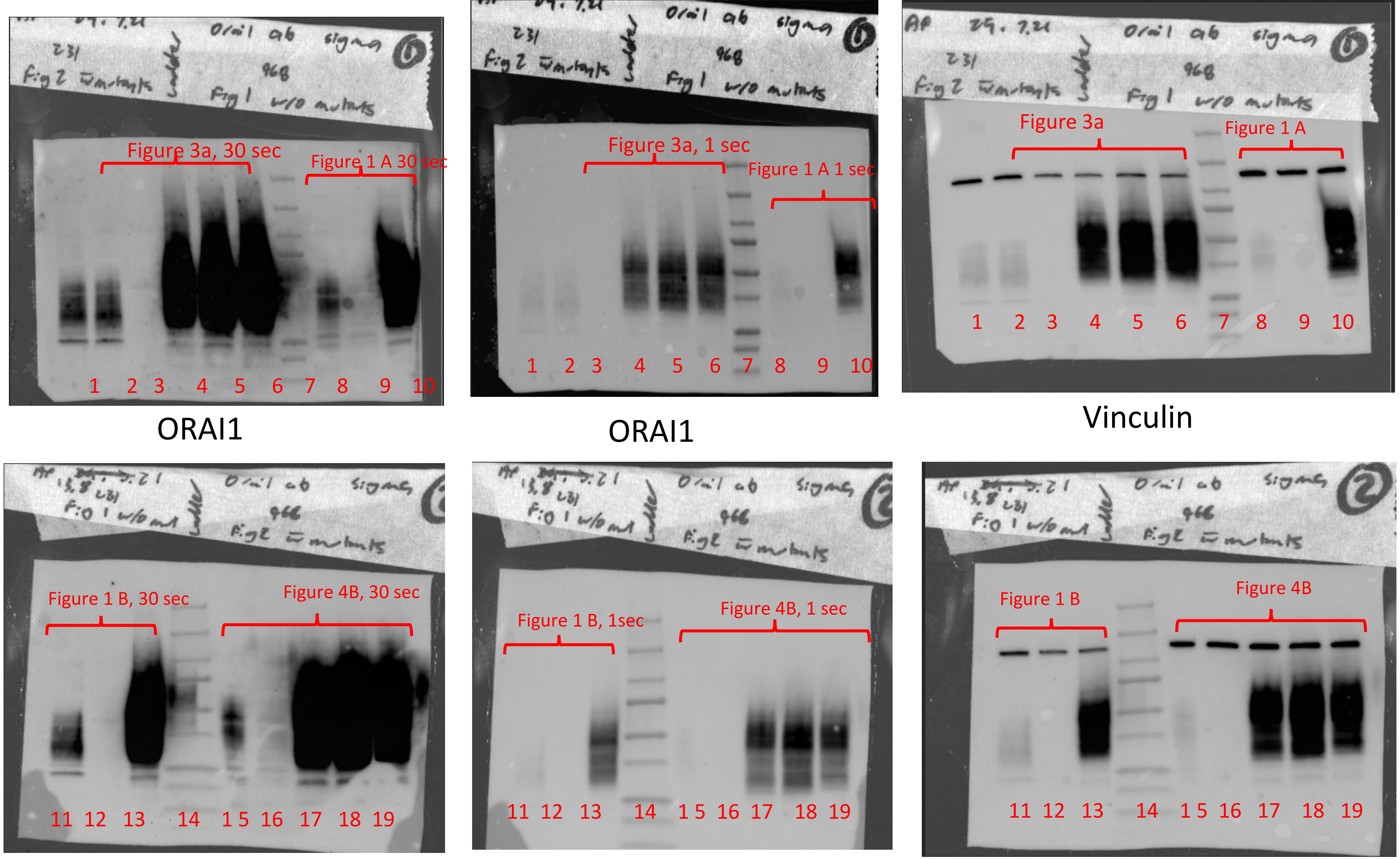

1. MDA231-CRISPR Scrambled
2. MDA231-parental
3. MDA231-ORAI1 KO
4. MDA231-ORAI1 KO + WT
5. MDA231-ORAI1 KO + E106Q
6. MDA231-ORAI1 KO + L273D
7. All blue prestained standards (Biorad)
8. MDA468-parental
9. MDA468-ORAI1 KO
10. MDA468-ORAI1 KO + WT

11. MDA231-parental
12. MDA231-ORAI1 KO
13. MDA231-ORAI1 KO + WT
14. All blue prestained standards (Biorad)
15. MDA468-parental
16. MDA468-ORAI1 KO
17. MDA468-ORAI1 KO + WT
18. MDA468-ORAI1 KO + E106Q
19. MDA468-ORAI1 KO + L273D

Supplementary Figure S2: Original western blots.
